# The Administration of Anæsthetics

**Published:** 1907-07-27

**Authors:** 


### THE ADMINISTRATION OF ANÆSTHETICS.

Sir,—In connection with the subject of anæsthetics, perhaps I may be permitted to say that my experience coincides with that of Dr. Hewitt regarding methylated chloroform, and I also have found it in no way inferior to ethylic chloroform. The matter was brought under my notice in a very practical manner some two years ago. Owing to financial stress, the Committee at a hospital where I was resident medical officer decided that methylated chloroform should be used instead of ethylic chloroform, and so it came to pass that during the first four months of my tenure of office I was using ethylic chloroform, and during the last eight months methylated chloroform. So far as could be roughly determined, there was no difference between the two.

The subject of the administration of anæsthetics is of paramount importance to resident medical officers, as in many hospitals they will find this is included among their duties. "Practice makes perfection," and it is only by practice that one can attain to any degree of confidence in giving anæsthetics. Everyone before qualifying should be compelled to attend a course of lectures on the subject, and should be made to give anæsthetics a certain number of times under skilled supervision. It is only within the last year or so that such a course has been made compulsory at the school where I was trained, and although it is a pity to have to add to the present heavy curriculum there can be no doubt that this addition is a proper one. It is impossible to learn how to give anæsthetics properly from the benches of an operating theatre or by reading text-books; indeed, I think the descriptions in some books are calculated unduly to alarm the uninitiated. When one considers that anyone who is going to engage in active medical practice is bound to have some anæsthetic work, is it not reasonable to ask that he should have some training in that branch of work before he qualifies?

There is no doubt that many of the fatalities under anæsthetics occur in inexperienced hands, and if that factor could be eliminated, the mortality would be greatly reduced, as the percentage of unavoidable (for there are cases in which a fatal termination will occur no matter how experienced the anæsthetist is) fatalities is very small. Dr. Hewitt's suggestion that there should be more resident anæsthetist appointments is an admirable one, and well worth earnest consideration. If, however, training in anæsthetic work were made compulsory for all medical qualifications, then one might hope to see some diminution in the list of fatalities, and the accusation of the totally inexperienced undertaking the work could not be made.

I am, etc.,

Alpha.

The Treasurers of the Royal National Orthopædic Hospital have received notice of a legacy of £300 under the will of the late Mr. Howard Pane.

